# Supplementary figures and images for: The comparative plastisphere microbial community profile at Kung Wiman beach unveils potential plastic-specific degrading microorganisms
Source: PeerJ. 2024 Apr 5;12:e17165. doi: 10.7717/peerj.17165 (PMC11000645; doi:10.7717/peerj.17165)

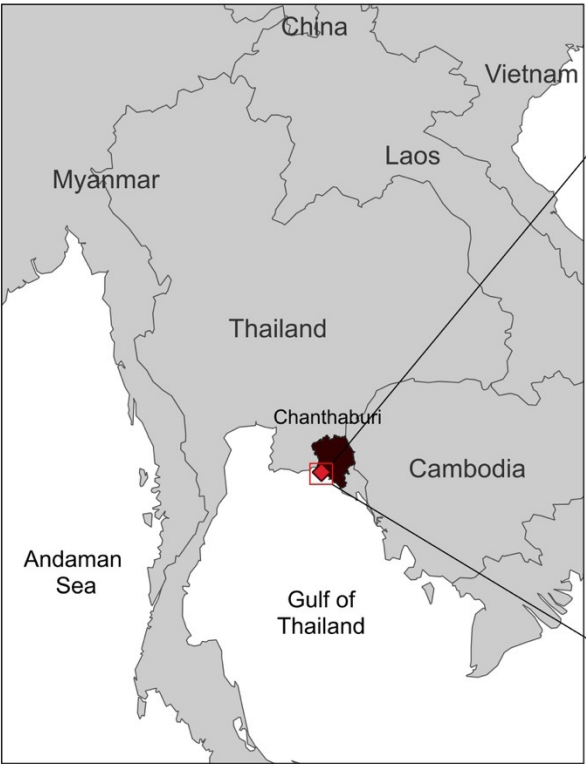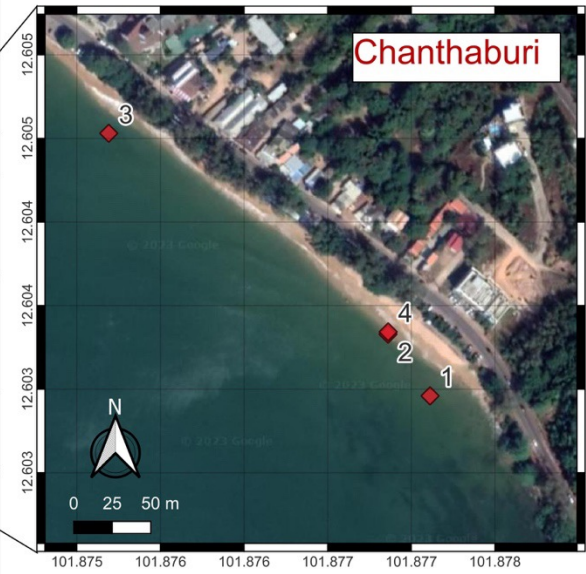

Supplement: Supplemental Information 1 [file peerj-12-17165-s001.pdf]

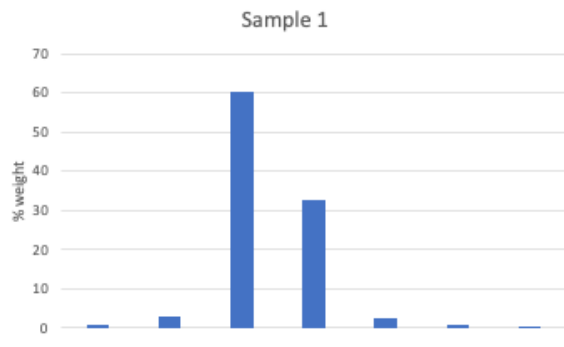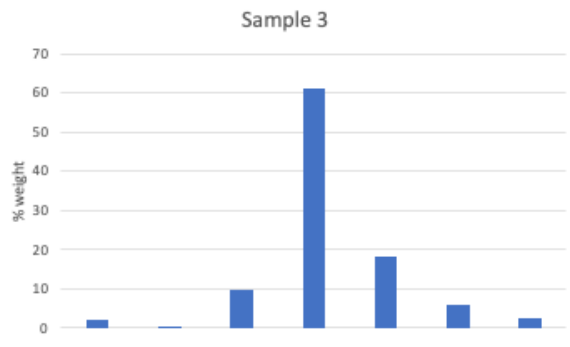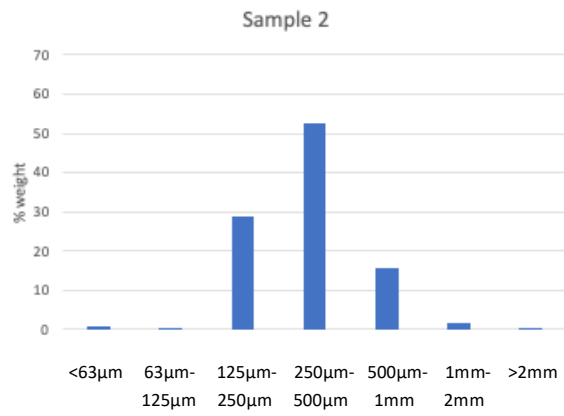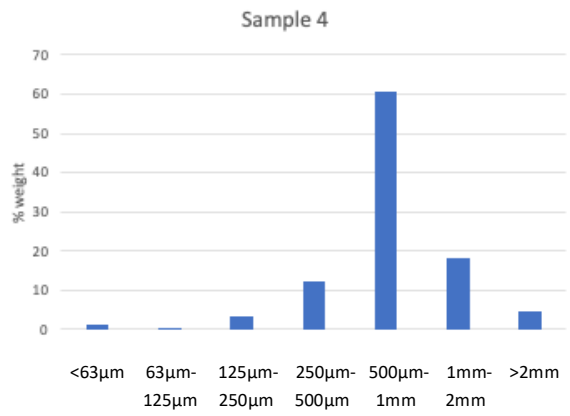

Supplement: Supplemental Information 2 [file peerj-12-17165-s002.pdf]

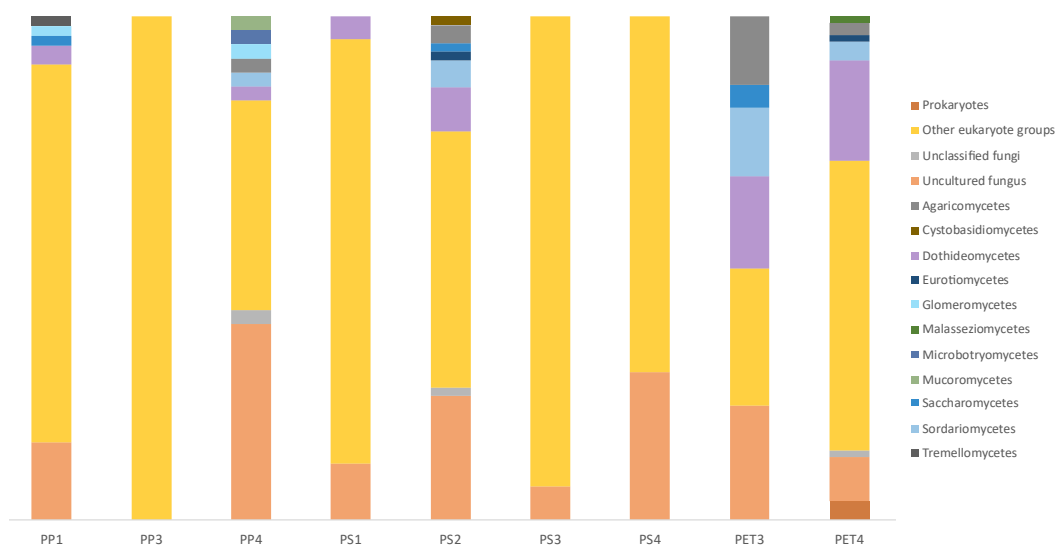

Supplement: Supplemental Information 4 [file peerj-12-17165-s004.pdf]
