# Supplementary material for: The comparative plastisphere microbial community profile at Kung Wiman beach unveils potential plastic-specific degrading microorganisms
Source: PeerJ. 2024 Apr 5;12:e17165. doi: 10.7717/peerj.17165 (PMC11000645; doi:10.7717/peerj.17165)

(1A) sample code 1.3

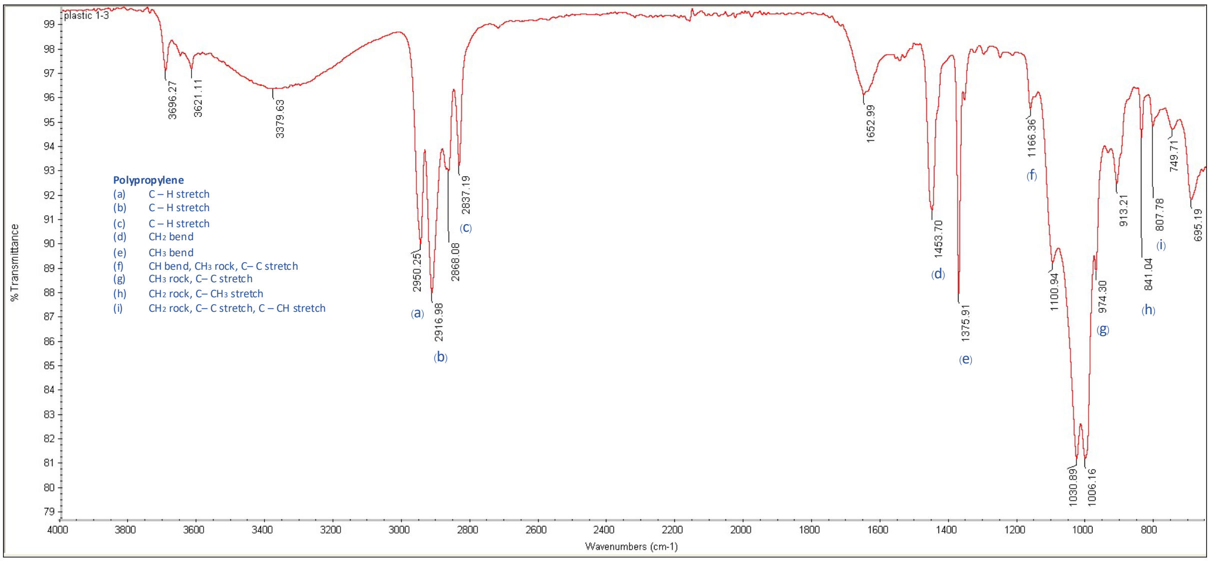

(2A) sample code 1.4

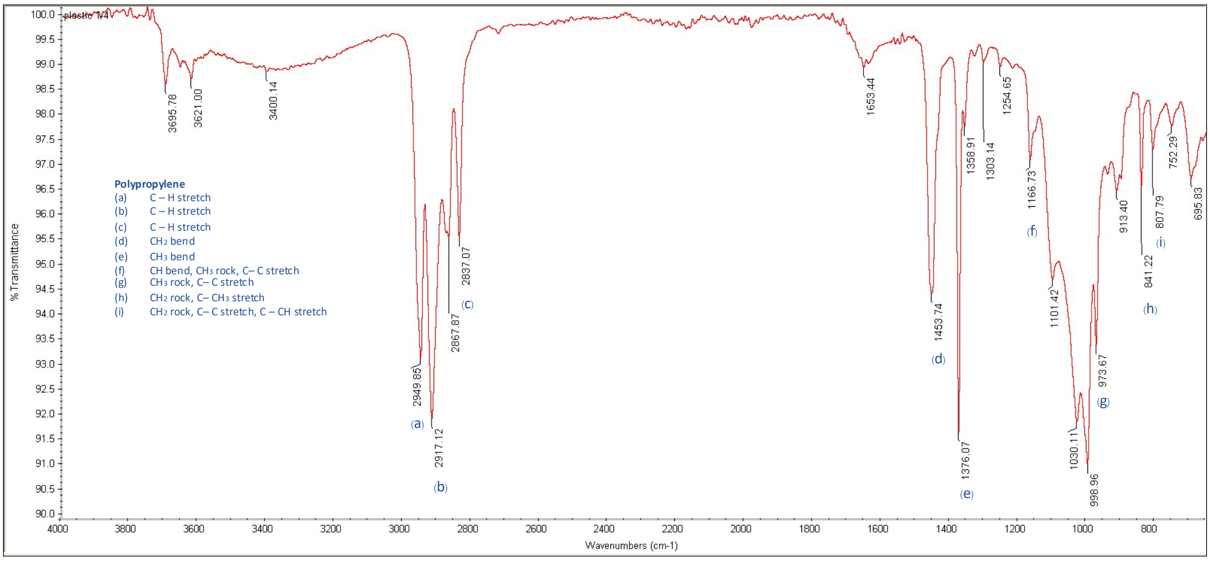

(3A) sample code 4.1

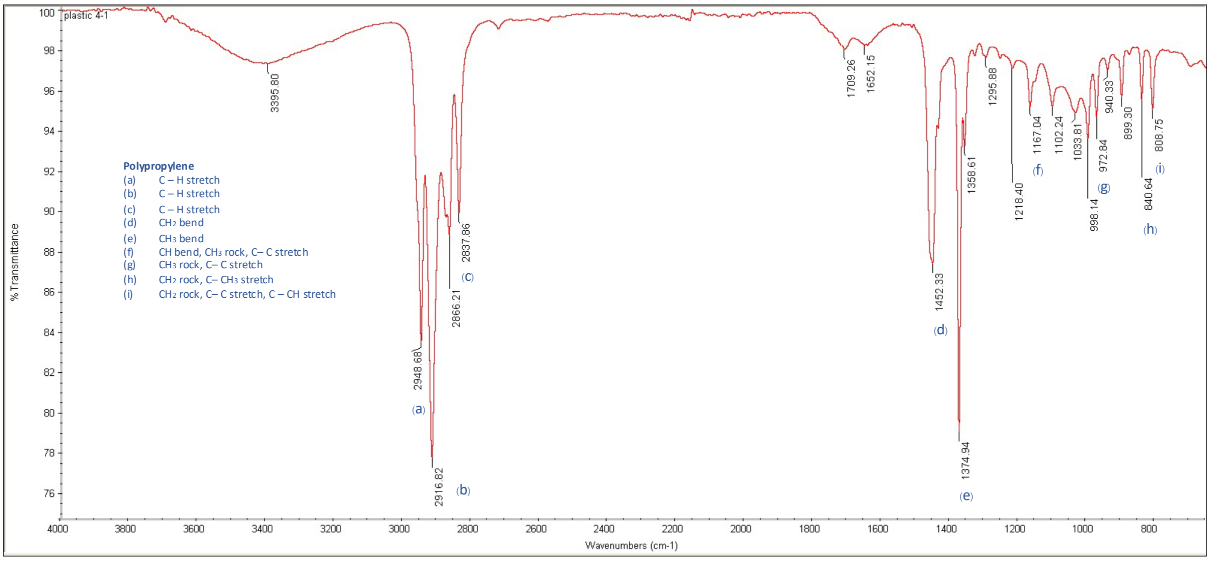

(4A) sample code 4.2

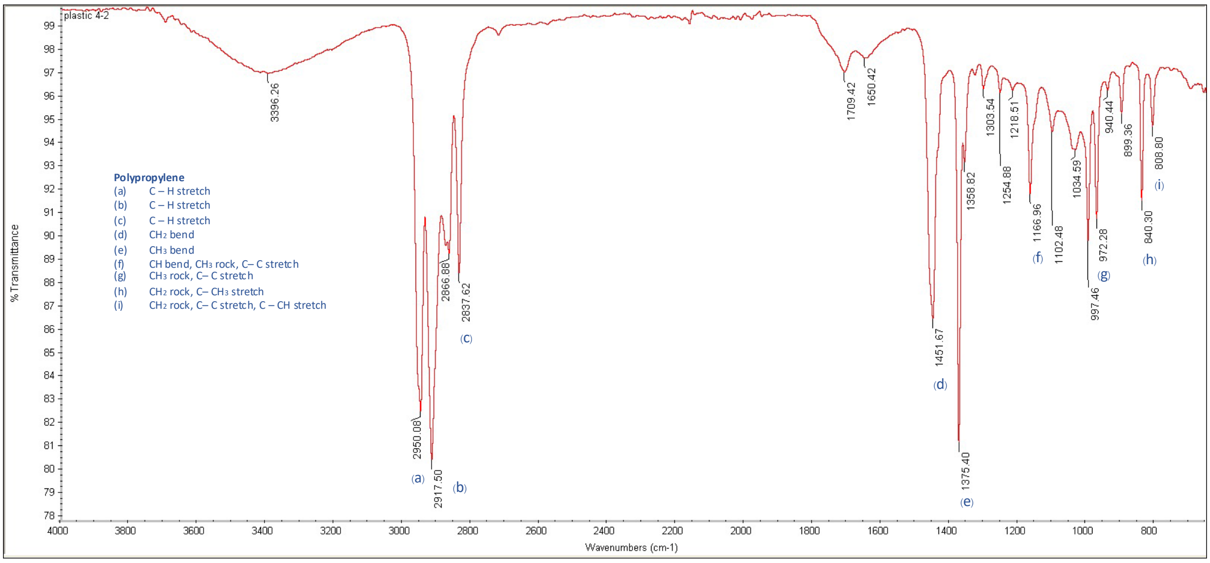

(5A) sample code 4.4

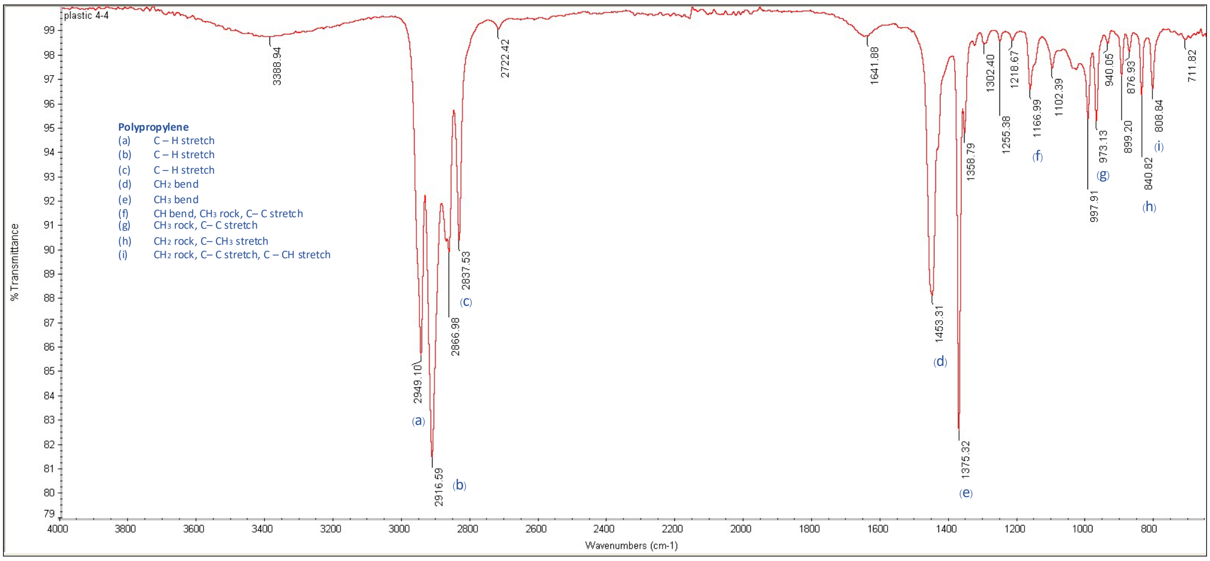

(1B) sample code 2.1

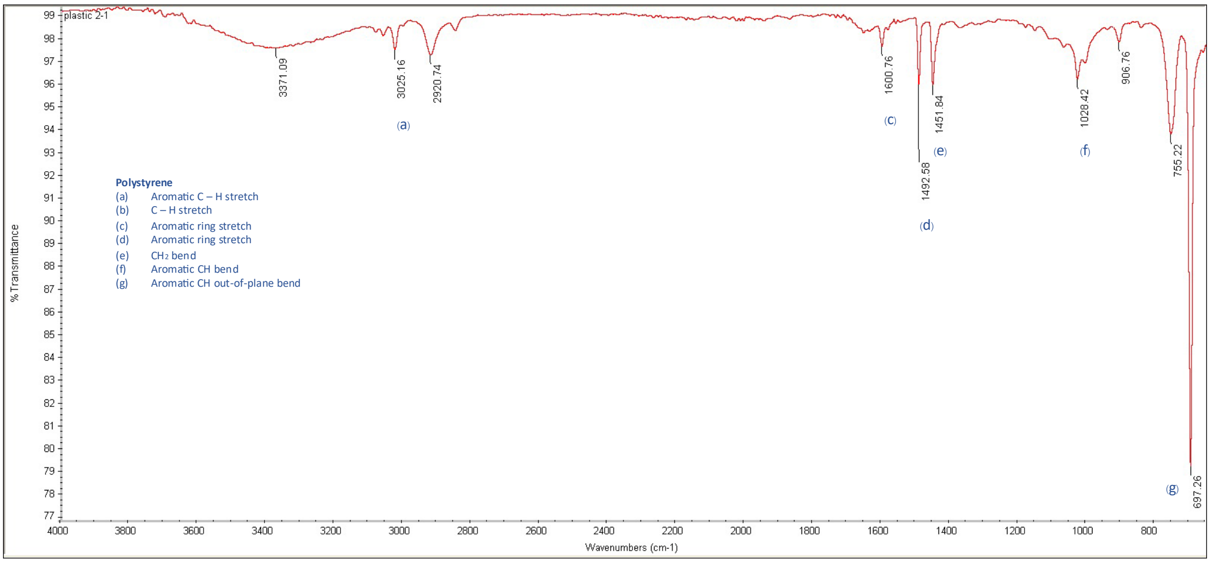

(2B) sample code 2.2

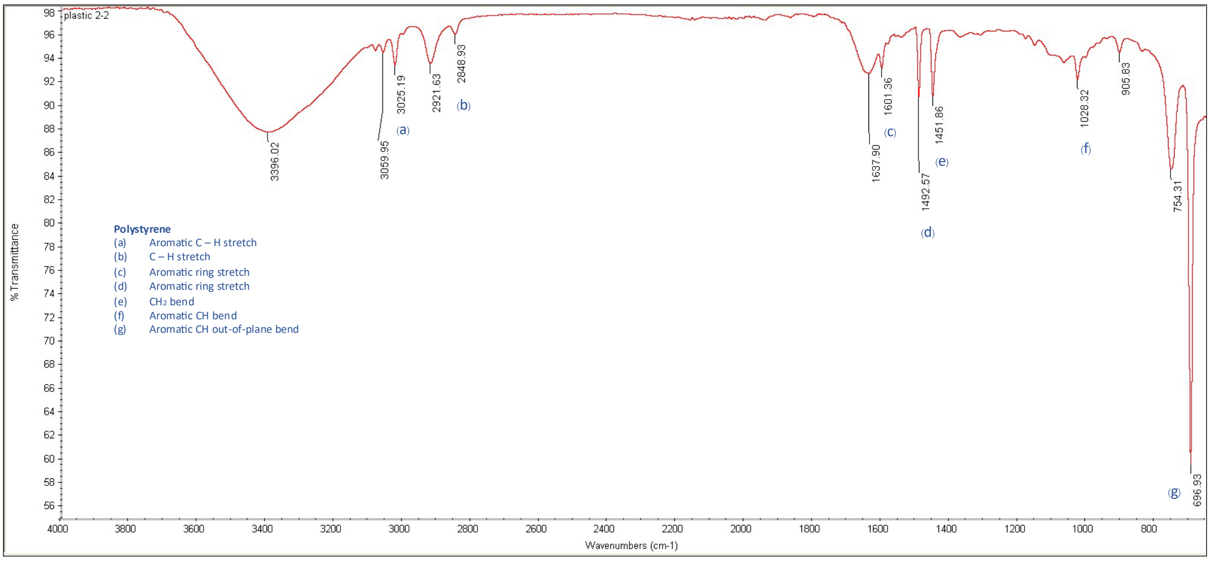

(3B) sample code 2.3

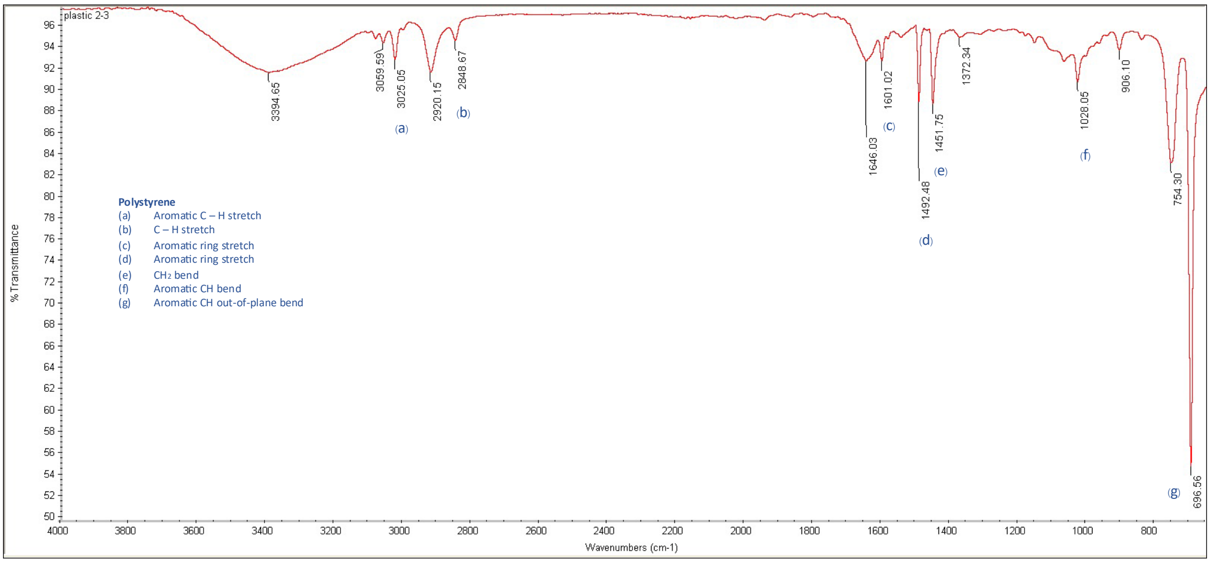

(4B) sample code 2.4

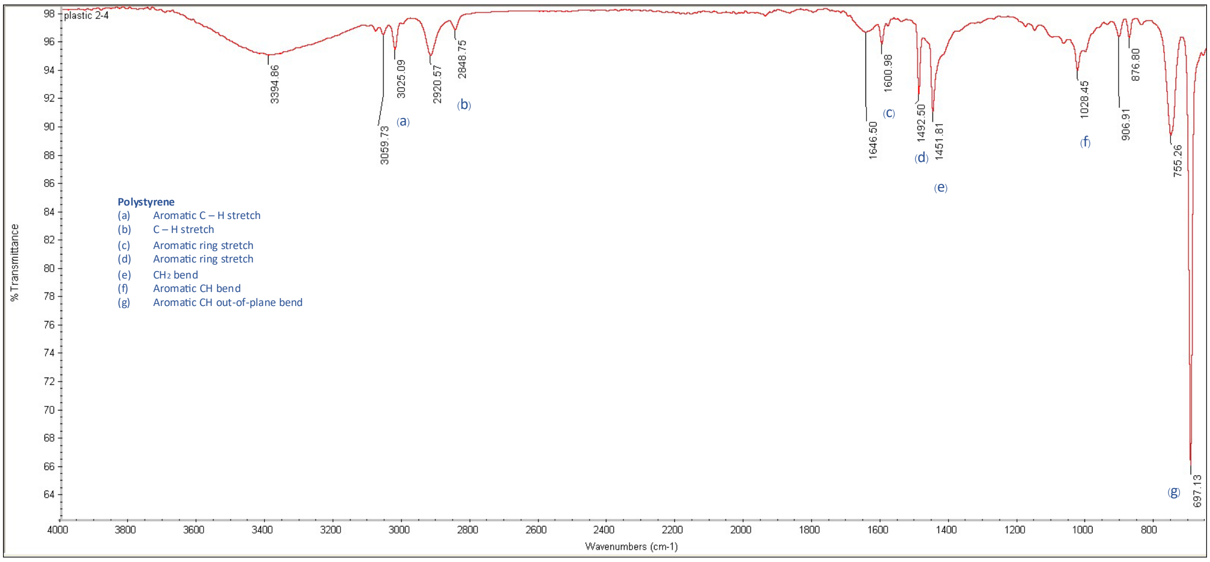

(1C) sample code 3.1

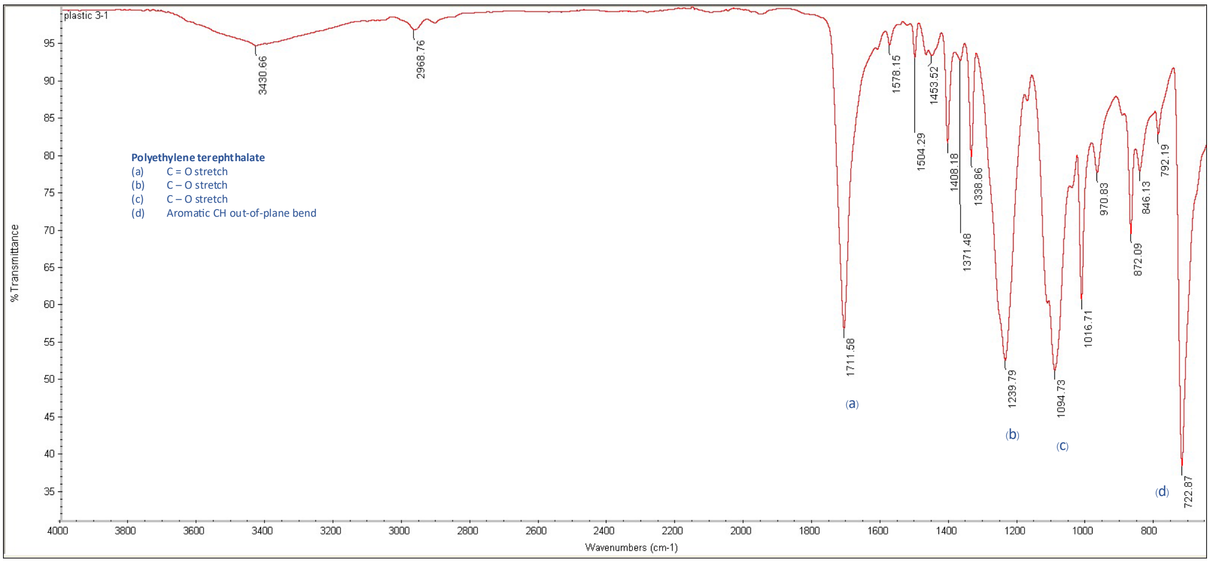

(2C) sample code 3.2

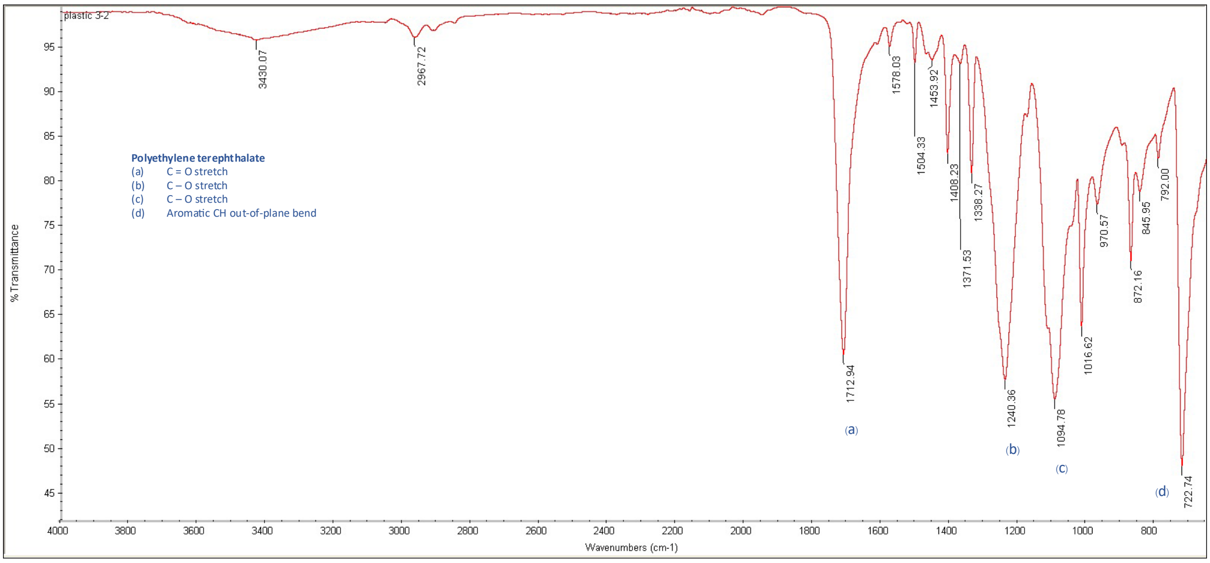

(3C) sample code 3.4

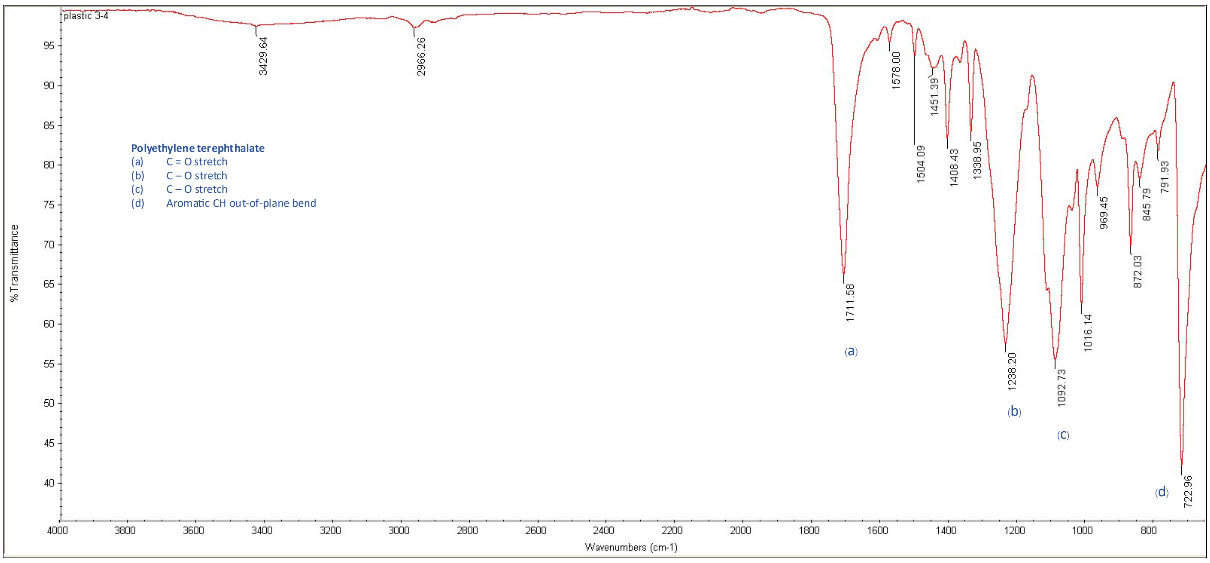

(4C) sample code 4.3

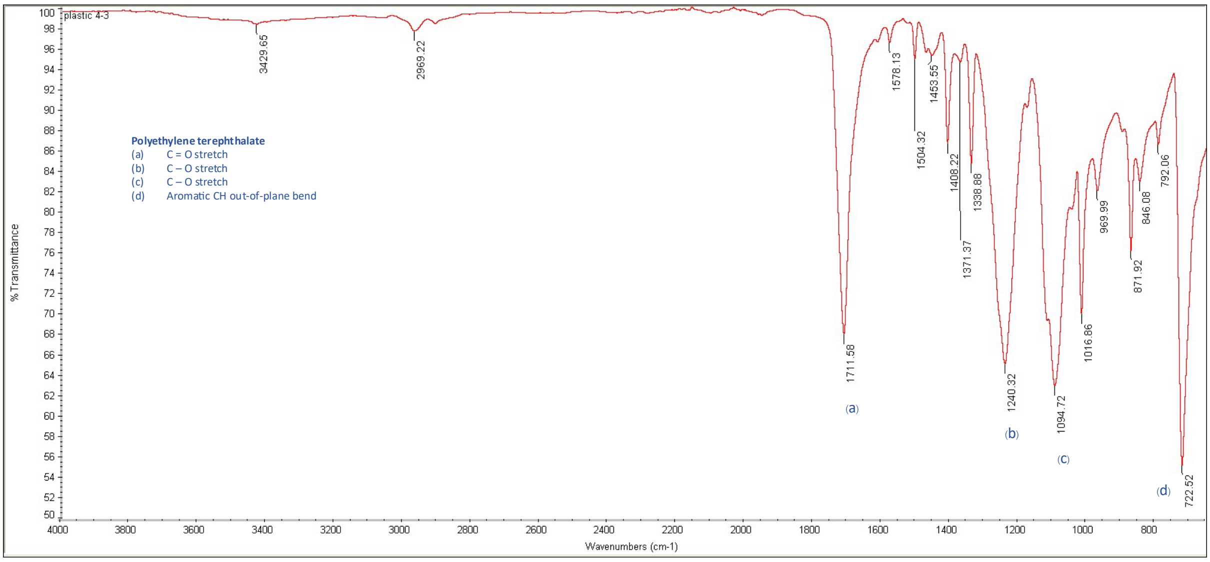

(1D) sample code 1.2

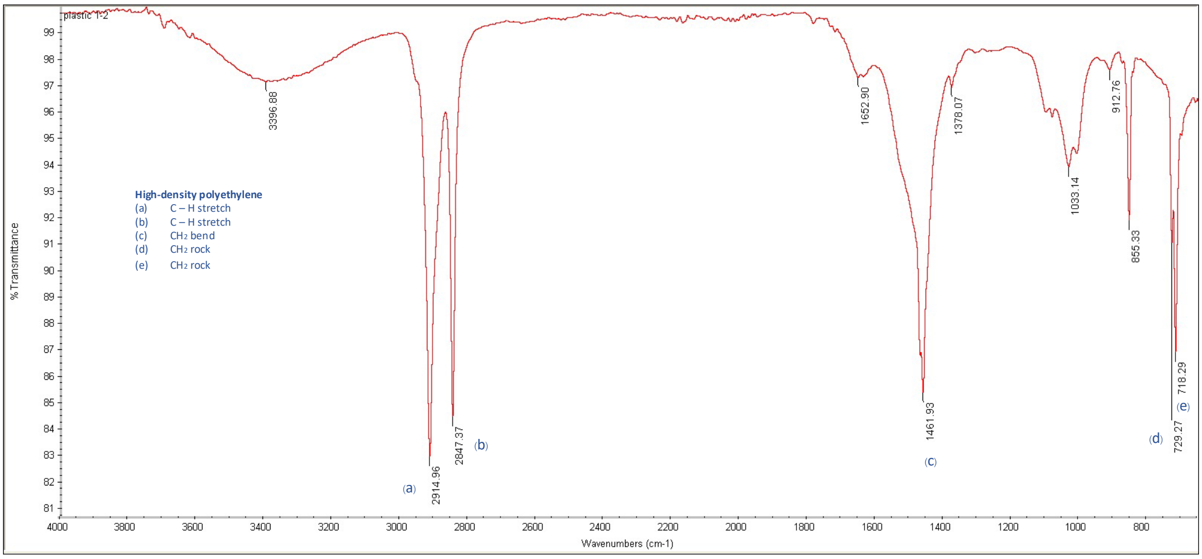

(2D) sample code 3.3

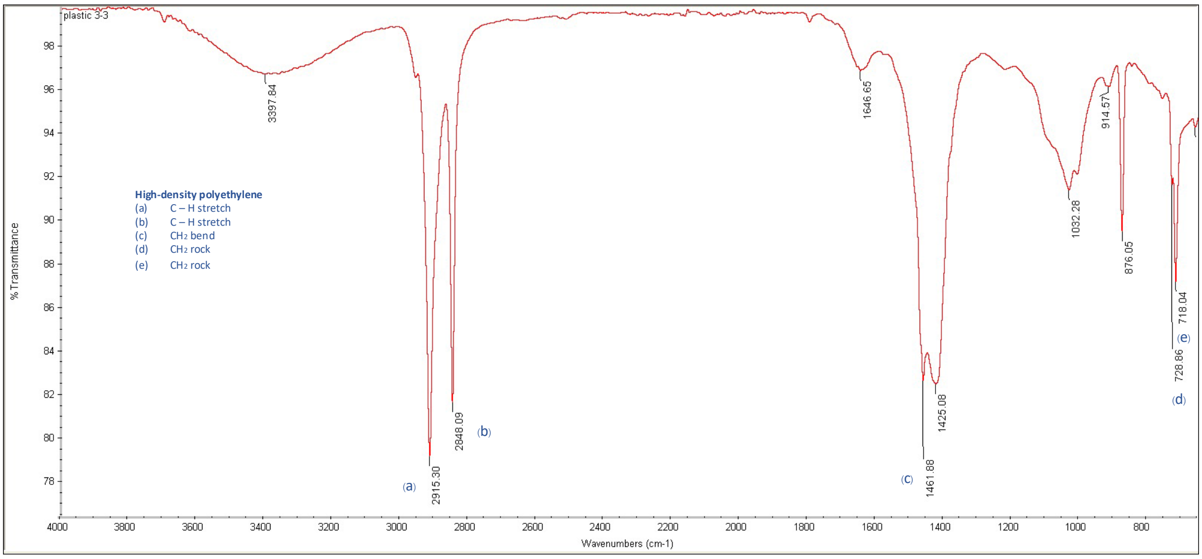

(E) sample code 1.1

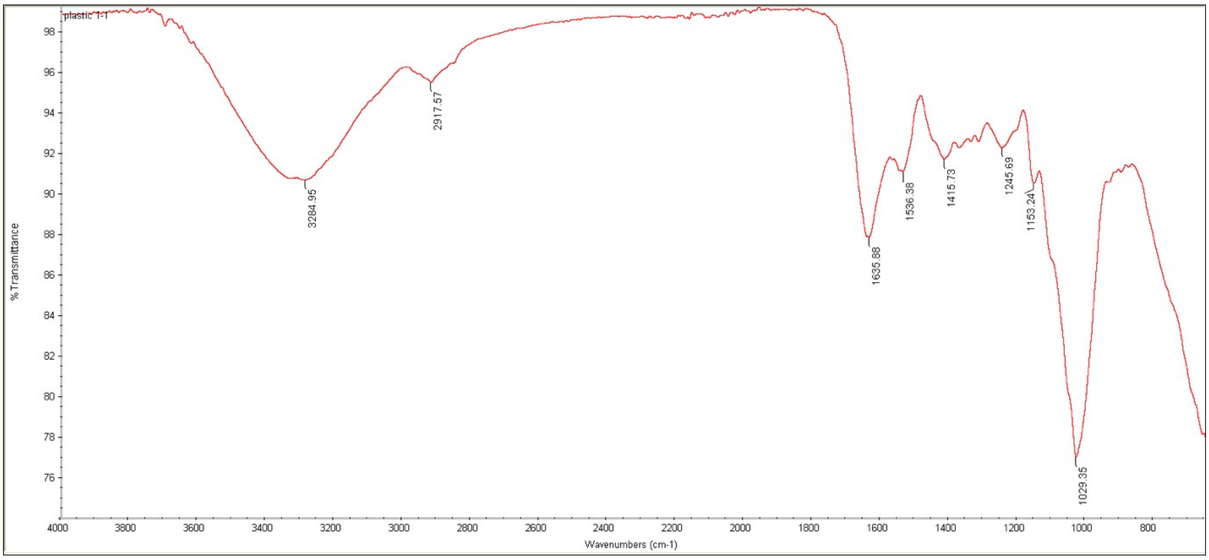

Supplement: Supplemental Information 3 — (1-5A) plastic samples identified as polypropylene (sample code 1.3, 1.4, 4.1, 4.2, and 4.4), (1-5B) polystyrene (sample codes: 2.1, 2.2, 2.3, and 2.4), (1-4C) polyethylene terephthalate (sample codes: 3.1, 3.2, 3.4, and 4.3), (1-2D) high-density polyethylene (sample codes: 1.2 and 3.3), and (E) the unidentified plastic (sample code: 1.1). [file peerj-12-17165-s003.pdf]
