# Supplementary material for: The comparative plastisphere microbial community profile at Kung Wiman beach unveils potential plastic-specific degrading microorganisms
Source: PeerJ. 2024 Apr 5;12:e17165. doi: 10.7717/peerj.17165 (PMC11000645; doi:10.7717/peerj.17165)
Supplement: Supplemental Information 5 [file peerj-12-17165-s005.docx]

| **Sample** | **Replicate** | **Dry weight (g)** | **The weight percentage of organic carbon (%)** | **The average weight percentage of organic carbon (% Carbon)** |
| --- | --- | --- | --- | --- |
| Sand 1 | 1 | 1.0105 | 0.10 | 0.09 |
|  | 2 | 1.2000 | 0.09 |  |
| Sand 2 | 1 | 1.1475 | 0.03 | 0.04 |
|  | 2 | 1.3390 | 0.06 |  |
| Sand 3 | 1 | 1.4028 | 0.03 | 0.04 |
|  | 2 | 1.1272 | 0.05 |  |
| Sand 4 | 1 | 1.1980 | 0.07 | 0.08 |
|  | 2 | 1.1648 | 0.09 |  |
