# Supplementary material for: The comparative plastisphere microbial community profile at Kung Wiman beach unveils potential plastic-specific degrading microorganisms
Source: PeerJ. 2024 Apr 5;12:e17165. doi: 10.7717/peerj.17165 (PMC11000645; doi:10.7717/peerj.17165)
Supplement: Supplemental Information 6 [file peerj-12-17165-s006.docx]

| **Plastic samples code** | **Plastic type** | **Plastic samples with a scale** |
| --- | --- | --- |
| 1.1 | unidentified | 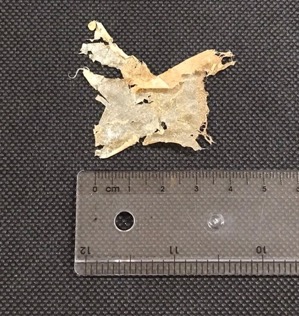 |
| 1,2 | HDPE | 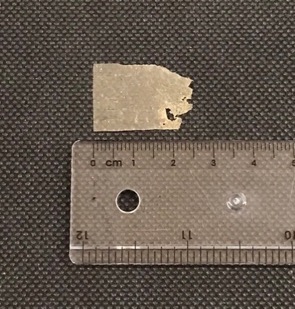 |
| 1.3 | PP | 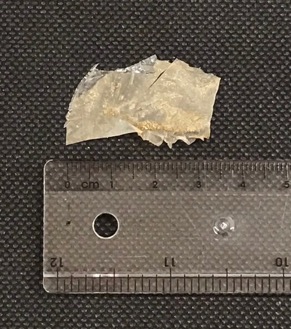 |
| 1.4 | PP | 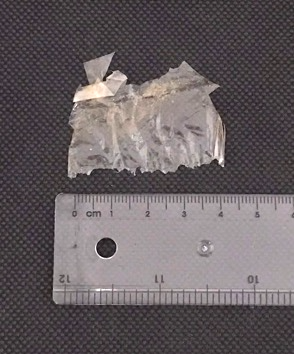 |
| 2.1 | PS | 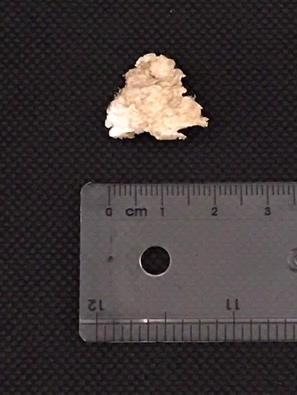 |
| 2.2 | PS | 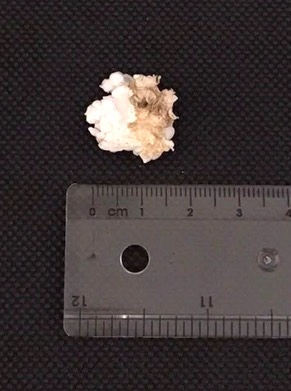 |
| 2.3 | PS | 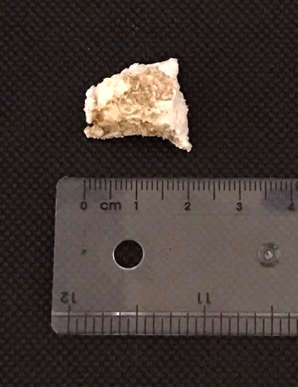 |
| 2.4 | PS | 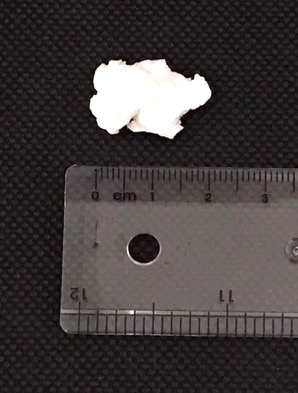 |
| 3.1 | PET | 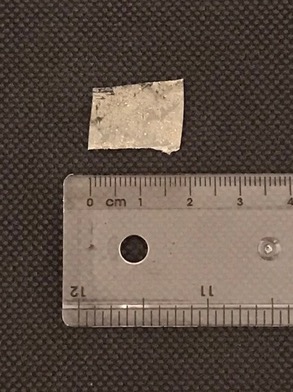 |
| 3.2 | PET | 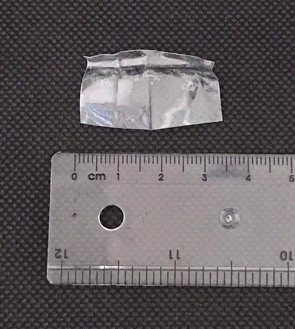 |
| 3.3 | HDPE | 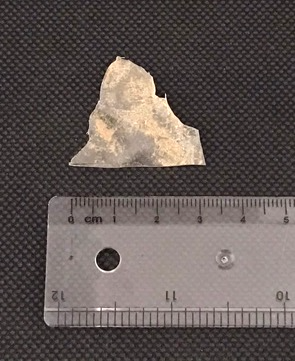 |
| 3.4 | PET | 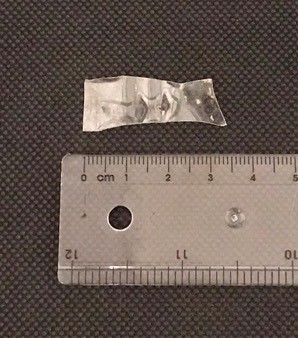 |
| 4.1 | PP | 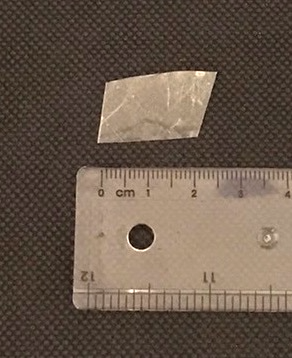 |
| 4.2 | PP | 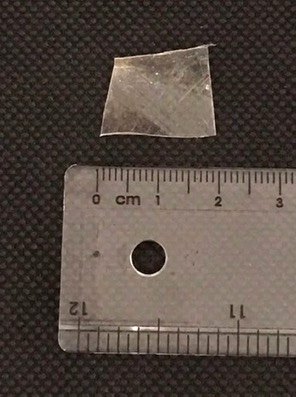 |
| 4.3 | PET | 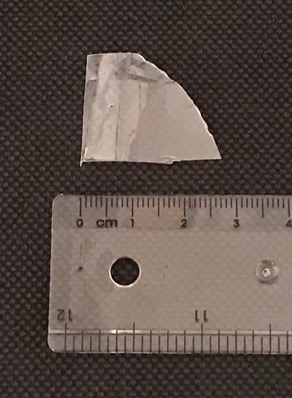 |
| 4.4 | PP | 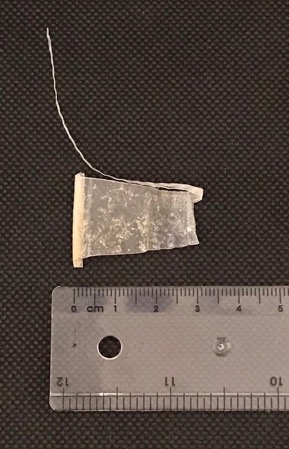 |
