# Supplementary material for: The comparative plastisphere microbial community profile at Kung Wiman beach unveils potential plastic-specific degrading microorganisms
Source: PeerJ. 2024 Apr 5;12:e17165. doi: 10.7717/peerj.17165 (PMC11000645; doi:10.7717/peerj.17165)
Supplement: Supplemental Information 7 — (A) PP, (B) PS, (C) PET, and (D) sand samples. [file peerj-12-17165-s007.docx]

(A)

| Strain code | Species | Accession number | % Similarity | Length (bp) |
| --- | --- | --- | --- | --- |
| PP 1 | *Marinobacter salsuginis*  SD-14B^T^ | EF028328 | 98.04 | 1,379 |
| PP 2 | *Pseudoalteromonas aliena*  KMM 3562^T^ | AY387858 | 98.75 | 1,445 |
| PP 3 | *Pseudoalteromonas carrageenovora*  IAM 12662^T^ | X82136 | 99.56 | 1,167 |
| PP 4 | *Salipiger pacificus*  DSM 26894^T^ | jgi.1055276 | 99.25 | 1,327 |
| PP 5 | *Planococcus plakortidis*  DSM 23997^T^ | CP016539 | 99.31 | 1,464 |
| PP 6 | *Planococcus maritimus*  DSM 17275^T^ | CP016538 | 99.57 | 1,403 |
| PP 7 | *Psychrobacter maritimus*  Pi2-20^T^ | AJ609272 | 99.78 | 1,387 |
| PP 8 | *Psychrobacter maritimus*  Pi2-20^T^ | AJ609272 | 99.14 | 1,396 |

(B)

| Strain code | Species | Accession number | % Similarity | Length (bp) |
| --- | --- | --- | --- | --- |
| PS 1 | *Shewanella baltica*  NCTC 10735 ^T^ | AJ000214 | 98.56 | 1,389 |
| PS 2 | *Pseudoalteromonas carrageenovora*  IAM 12662^T^ | X82136 | 92.39 | 1,106 |
| PS 3 | *Pseudoalteromonas tetraodonis*  GFC ^T^ | CP011041 | 99.51 | 1.435 |
| PS 4 | *Pseudoalteromonas tetraodonis*  GFC ^T^ | CP011041 | 99.50 | 1,424 |
| PS 5 | *Planococcus maritimus*  DSM 17275 ^T^ | CP016538 | 99.58 | 1,422 |
| PS 6 | *Exiguobacterium profundum*  10C ^T^ | AY818050 | 99.93 | 1,429 |

(C)

| Strain code | Species | Accession number | % Similarity | Length (bp) |
| --- | --- | --- | --- | --- |
| PET 1 | *Marinomonas piezotolerans*  YLB-05 ^T^ | QKRA01000025 | 99.64 | 1,381 |
| PET 2 | *Cobetia marina*  JCM 21022 ^T^ | CP017114 | 99.93 | 1,410 |
| PET 3 | *Planococcus maritimus*  DSM 17275 ^T^ | CP016538 | 99.79 | 1,405 |
| PET 4 | *Jonesia quinghaiensis*  DSM 15701 ^T^ | AJ626896 | 99.20 | 1,383 |
| PET 5 | *Exiguobacterium profundum*  10C ^T^ | AY818050 | 98.36 | 1,523 |
| PET 6 | UN |  |  |  |
| PET 7 | UN |  |  |  |
| PET 8 | *Bacillus siamensis*  KCTC 13613 ^T^ | AJVF01000043 | 99.85 | 1,311 |

Key: UN, unidentified.

(D)

| Strain code | Species | Accession number | % Similarity | Length (bp) |
| --- | --- | --- | --- | --- |
| Sand 1 | *Marinobacter salsuginis*  SD-14B^T^ | EF028328 | 100 | 1,381 |
| Sand 2 | *Bacillus altitudinis*  41KF2b^T^ | ASJC01000029 | 100 | 1,410 |
| Sand 3 | *Kocuria rosea*  DSM 20447^T^ | X87756 | 99.05 | 1,405 |
| Sand 4 | *Psychrobacter maritimus*  Pi2-20^T^ | AJ609272 | 99.86 | 1,383 |
| Sand 5 | *Halomonas meridiana*  DSM 5425^T^ | AJ306891 | 99.78 | 1,523 |
