# Supplementary material for: The comparative plastisphere microbial community profile at Kung Wiman beach unveils potential plastic-specific degrading microorganisms
Source: PeerJ. 2024 Apr 5;12:e17165. doi: 10.7717/peerj.17165 (PMC11000645; doi:10.7717/peerj.17165)
Supplement: Supplemental Information 8 — (A) PP, (B) PS, (C) PET, and (D) sand samples. [file peerj-12-17165-s008.docx]

(A)

| Strain code | Species | UNITE fungi reference | % Similarity | Length (bp) |
| --- | --- | --- | --- | --- |
| PP 1 | *Aspergillus foetidus*  ATCC16878 | UDB035087 | 99.82 | 583 |
| PP 2 | *Aspergillus niger*  3114-2T42 | UDB0799176 | 88.19 | 554 |
| PP 3 | *Aspergillus foetidus*  ATCC16878 | UDB035087 | 99.47 | 581 |
| PP 4 | *Aspergillus foetidus*  ATCC16878 | UDB035087 | 99.82 | 580 |
| PP 5 | *Aspergillus versicolor*  UTF96-121 | UDB028445 | 95.93 | 552 |
| PP 6 | *Pestalotiopsis montellicoides* ATCC62507 | UDB035208 | 86.15 | 535 |
| PP 7 | *Paecilomyces subglobosus* ATCC16492 | UDB035103 | 97.23 | 583 |
| PP 8 | *Absidia idahoensis*  ATCC76237 | UDB035468 | 87.18 | 556 |
| PP 9 | *Penicillium citrinum* | UDB023806 | 100 | 539 |
| PP 10 | *Penicillium lapatayae* ATCC60197 | UDB035204 | 92.61 | 544 |

(B)

| Strain code | Species | UNITE fungi number | % Similarity | Length (bp) |
| --- | --- | --- | --- | --- |
| PS 1 | *Penicillium asturianum* ATCC42226 | UDB035150 | 99.24 | 524 |
| PS 2 | *Aspergillus niger*  3114-2T42 | UDB0799176 | 89.04 | 546 |
| PS 3 | *Aspergillus niger*  3114-2T42 | UDB0799176 | 88.19 | 550 |
| PS 4 | *Aspergillus foetidus*  ATCC16878 | UDB035087 | 99.82 | 582 |
| PS 5 | *Aspergillus versicolor*  UTF96-121 | UDB028445 | 95.74 | 551 |
| PS 6 | *Pestalotiopsis montellicoides* ATCC62507 | UDB035208 | 86.15 | 537 |
| PS 7 | *Cochliobolus dactyloctenii* ATCC48875 | UDB035308 | 98.12 | 549 |
| PS 8 | UN |  |  |  |
| PS 9 | *Absidia idahoensis*  ATCC76237 | UDB035468 | 87.18 | 532 |
| PS 10 | UN |  |  |  |
| PS 11 | *Diplodia agrifolia*  ATCC-MYA-4895 | UDB035100 | 96.51 | 525 |

UN; Unidentified

(C)

| Strain code | Species | UNITE fungi number | % Similarity | Length (bp) |
| --- | --- | --- | --- | --- |
| PET 1 | *Aspergillus niger*  3114-2T42 | UDB0799176 | 88.19 | 555 |
| PET 2 | *Aspergillus foetidus*  ATCC16878 | UDB035087 | 99.64 | 582 |
| PET 3 | *Aspergillus flavus*  3111-2A10 | UDB0799174 | 96.84 | 574 |
| PET 4 | *Fusarium sp.*  3187-2F42 | UDB0799228 | 99.80 | 525 |
| PET 5 | *Cochliobolus neergaardii* ATCC76868 | UDB035514 | 96.33 | 548 |
| PET 6 | *Absidia idahoensis*  ATCC76237 | UDB035468 | 87.18 | 565 |
| PET 7 | *Petromyces albertensis* ATCC58745 | UDB035114 | 90.38 | 585 |
| PET 8 | UN |  |  |  |
| PET 9 | UN |  |  |  |

Key: UN, unidentified

(D)

| Strain code | Species | UNITE fungi number | % Similarity | Length (bp) |
| --- | --- | --- | --- | --- |
| Sand 1 | *Penicillium lapatayae* ATCC60197 | UDB035204 | 94.31 | 560 |
| Sand 2 | *Aspergillus foetidus*  ATCC16878 | UDB035087 | 99.82 | 578 |
| Sand 3 | *Aspergillus versicolor*  UTF96-121 | UDB028445 | 95.44 | 543 |
| Sand 4 | *Aspergillus flavus*  3111-2A10 | UDB0799174 | 99.82 | 579 |
| Sand 5 | *Fusarium sp.*  3187-2F42 | UDB0799228 | 99.80 | 525 |
| Sand 6 | UN |  |  |  |
| Sand 7 | *Aspergillus versicolor*  UTF96-121 | UDB028445 | 95.99 | 546 |
| Sand 8 | UN |  |  |  |

Key: UN, unidentified.
