# Supplementary material for: The comparative plastisphere microbial community profile at Kung Wiman beach unveils potential plastic-specific degrading microorganisms
Source: PeerJ. 2024 Apr 5;12:e17165. doi: 10.7717/peerj.17165 (PMC11000645; doi:10.7717/peerj.17165)
Supplement: Supplemental Information 9 [file peerj-12-17165-s009.docx]

| **Isolated plastic-degrading bacteria** | **Replicate 1** | | | **Replicate 2** | | | **Replicate 3** | | | **Average of HC value** |
| --- | --- | --- | --- | --- | --- | --- | --- | --- | --- | --- |
|  | **Colony size** | **Clear zone size** | **HC value** | **Colony size** | **Clear zone size** | **HC value** | **Colony size** | **Clear zone size** | **HC value** |  |
|  | **(cm)** | **(cm)** |  | **(cm)** | **(cm)** |  | **(cm)** | **(cm)** |  |  |
| *Salipiger* sp. PP4 | 1.0 | 1.4 | 1.40 | 0.9 | 1.4 | 1.56 | 0.9 | 1.4 | 1.56 | 1.51 ± 0.09 |
| *Planococcus* sp. PP5 | 0.7 | 1.2 | 1.71 | 0.7 | 1.2 | 1.71 | 0.8 | 1.2 | 1.50 | 1.64 ± 0.12 |
| *Psychrobacter* sp. PP7 | 0.9 | 1.3 | 1.44 | 0.7 | 1.0 | 1.43 | 1.0 | 1.3 | 1.30 | 1.39 ± 0.08 |
| *Shewanella* sp. PS1 | 1.0 | 1.2 | 1.20 | 0.8 | 1.0 | 1.25 | 0.8 | 1.0 | 1.25 | 1.23 ± 0.03 |
| *Planococcus* sp. PS5 | 0.8 | 1.0 | 1.25 | 0.8 | 1.2 | 1.50 | 0.8 | 1.2 | 1.50 | 1.42 ± 0.14 |
| *Jonesia* sp. PET4 | 0.7 | 1.2 | 1.71 | 0.7 | 1.0 | 1.43 | 0.9 | 1.3 | 1.44 | 1.53 ± 0.16 |
| *Bacillus* sp. PET8 | 5.1 | 5.2 | 1.02 | 5.3 | 5.5 | 1.04 | - | - | - | 1.03 ± 0.01 |
| *Bacillus* sp. Sand2 | 1.9 | 2.0 | 1.05 | 1.8 | 1.9 | 1.06 | 1.6 | 1.7 | 1.06 | 1.06 ± 0.01 |
| *Kocuria* sp. Sand3 | 0.7 | 1.0 | 1.43 | 0.7 | 1.2 | 1.71 | - | - | - | 1.57 ± 0.20 |
| *Psychrobacter* sp. Sand4 | NA | | | | | | | | | |

Key: NA, not available.
